# Safe resection margin in video-assisted left anterior and lingular segmentectomy for an impalpable lung nodule

**DOI:** 10.1016/j.ijscr.2019.04.048

**Published:** 2019-05-07

**Authors:** Kotaro Mizuno, Tadashi Sakane

**Affiliations:** a Department of Thoracic Surgery, Nagoya City East Medical Center, Japan; b Department of Oncology, Immunology and Surgery, Nagoya City University Graduate School of Medical Sciences, Japan

**Keywords:** Video-assisted thoracoscopic surgery, Segmentectomy, Minimally invasive surgery

## Abstract

**Introduction:**

The nodule located at the left anterior segment near the lingular segment is traditionally resected by left upper lobectomy. We performed video-assisted thoracoscopic surgery (VATS) segmentectomy and could achieve a complete resection that is minimally invasive and oncologically sufficient.

**Presentation of case:**

An 82-year-old woman was found to have a nodule in the left anterior segment of the lung on chest computed tomography (CT). The nodule was 1.9 cm in size and strongly suspected to be lung carcinoma. No suspicious regions of metastasis were observed; thus, we diagnosed stage IA2 and decided to perform anterior and lingular segmentectomy by VATS.

**Discussion:**

Because of the location, the tumor is traditionally resected by left upper lobectomy. However, we planned a minimally invasive intervention and performed anterior and lingular segmentectomy by VATS using a CT-guided nodule marking prior to the surgery.

**Conclusion:**

This technique resulted in complete tumor resection with minimal adverse effects.

## Highlights

• This report is a rare case of left anterior and lingular segmentectomy by video-assisted thoracoscopic surgery.
• The preoperative CT-guided marking is useful for delineating safe margins in segmentectomy for invisible lesions.
• Thoracoscopic segmentectomy could achieve a complete resection that is minimally invasive and oncologically sufficient.

## 1 Introduction

Recently, VATS has been frequently performed and it contributes to the maintenance of postoperative quality of life. Furthermore, pulmonary segmentectomy is a useful method that can perform minimally invasive and oncologically sufficient for early stage lung cancer patients. The nodule located at the left anterior segment near the lingular segment, which is our case, is traditionally resected by left upper lobectomy. However, we planned a VATS segmentectomy to reduce the invasiveness to the patient. Segmentectomy has a risk of locoregional recurrence if a sufficient margin cannot be secured. We envisaged that a CT-guided nodule marking prior to VATS segmentectomy could achieve a complete resection that is minimally invasive and oncologically sufficient.

This case report has been written in line with the SCARE guidelines [1].

## 2 Presentation of case

### 2.1 Patient information

**Fig. 1:**
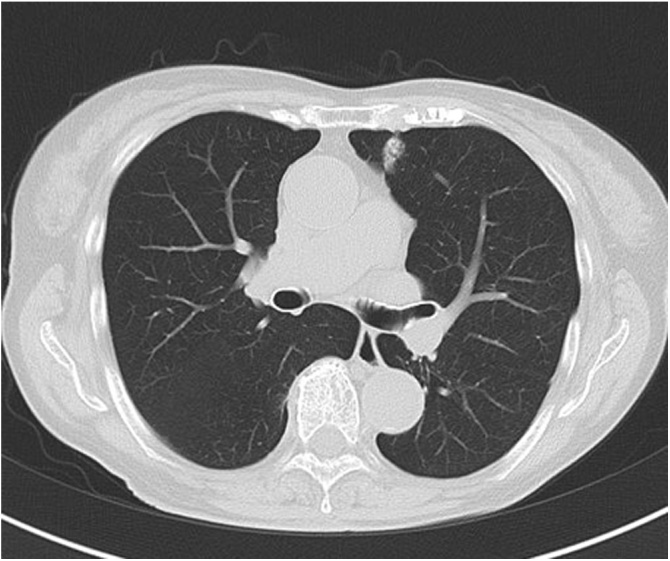
Chest computed tomography revealed a 1.9 × 1.1 cm nodule in the left anterior segment. The nodule was located near the lingular segment.

An 82-year-old woman being treated for pyelonephritis was referred to our hospital because of a nodule in the left anterior segment of the lung on chest CT. The nodule was part solid, 1.9 × 1.1 cm in size and reveal a pleural tag (Fig. 1). The maximum standardized uptake value of the tumor is 5.7 by 18F-fluorodeoxyglucose-positron emission tomography. The nodule was not confirmed by any histopathological examination; however, it was strongly suspected to be lung adenocarcinoma. Brain magnetic resonance imaging and abdominal CT revealed no obvious metastases. The clinical stage was T1bN0M0 stage IA2 as categorized by the UICC TNM Classification (8th edition). Spirometry demonstrated an FEV1.0 of 1.47 L and FEV1.0% of 53.4%. Moreover, the cardiac function was maintained to be normal.

We recommended the patient to undergo a lung resection for the diagnosis and treatment of the nodule, and she agreed. We believed that this lesion could be completely resected by anterior and lingular segmentectomy based on preoperative imaging diagnosis. However, if the nodule is not palpated, then a sufficient tumor margin may not be secured; thus, we decided to perform a CT-guided nodule marking prior to the surgery.

### 2.2 Preoperative CT-guided marking and operative techniques

**Fig. 2:**
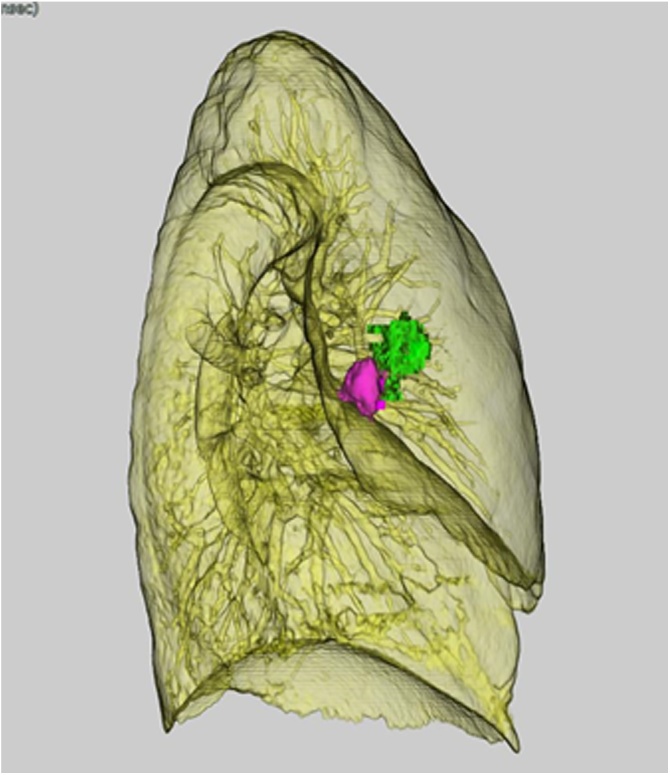
Three-dimensional construction image after a computed tomography-guided marking. Red shows a tumor, and green shows an injected site.

Prior to the surgery, she went to the CT room and was injected with indocyanine green under the visceral pleura near the tumor using a 23-G needle while checking the site using CT fluoroscopy (Fig. 2). When the marking was complete, she was immediately moved to the operation room.

The patient received epidural and general anesthesia. She was intubated with a double-lumen endotracheal tube and placed in a lateral decubitus position.

A utility incision (3.0 cm) was created in the seventh ICS on the posterior axillary line and was protected with a wound retractor XS (Medtronic Company). This utility incision was used to insert a thoracoscope and remove the resected specimen. Incisions for two 12-mm thoracoscopic port (Medtronic Company) were created in the fourth and sixth ICS on the anterior axillary line, which were used by a surgeon. In addition, a 1.0-cm incision was created in the sixth ICS under the scapula, which was used by an assistant.

**Fig. 3:**
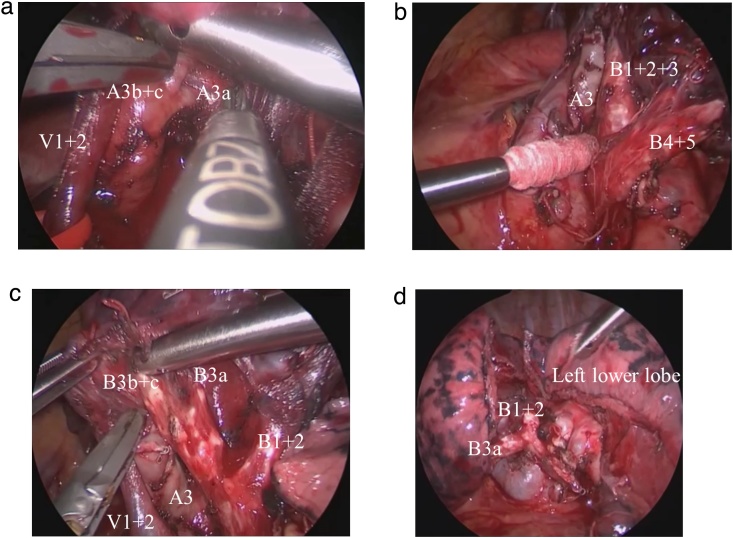
Video-assisted thoracoscopic left anterior and lingular segmentectomy. A: The anterior artery was separated into two branches. B: The lingular segmental bronchus was exposed. C: The anterior bronchus was exposed and separated into two branches. D: The intersegmental fissure was separated with endostaplers.

First, we confirmed an indocyanine green marking under the visceral pleura of the upper lobe. Based on the position of the marking, it was considered that the left anterior and lingular segmentectomy was possible as planned. We could not locate the tumor only by observing and palpation. The mediastinal pleura near the superior pulmonary vein were opened along the phrenic nerve, and the lingular vein was exposed. The inferior and superior lingular veins were separated from each other and resected after the ligation of the proximal end with a 3-0 silk suture. Subsequently, the anterior vein was exposed and separated into two branches (V3a and V3b + c) and the branches were resected after ligation with a 3-0 silk suture. The anterior artery was exposed and separated into two branches (A3a and A3b + c) by lifting the apicoposterior vein caudally (Fig. 3A). Then, only the A3b + c branch was transected using a 35-mm (white) endostapler (Johnson and Johnson Medical Company [JJMC]). The interlobar (11) lymph node was dissected after the lingular segmental bronchus and lower lobe bronchus were exposed and confirmed to be free of metastasis by intraoperative pathological diagnosis. The interlobar fissure between the lingular segment and the lower lobe was divided using a 60-mm (gold) endostapler (JJMC). The inferior lingular artery was exposed and resected after the ligation of the proximal end with a 3-0 silk suture. The interlobar fissure between the superior and lower lobes was divided using two 60-mm (gold and blue) endostaplers (JJMC). Subsequently, the superior lingular artery was ligated at the proximal end with a 3-0 silk suture and resected. The lingular segmental bronchus was exposed and transected using a 60-mm (gold) endostapler (JJMC) (Fig. 3B). The anterior bronchus was exposed and separated into two branches (B3a and B3b + c) (Fig. 3C). The B3b + c branch was resected after the ligation of the proximal end with a 1-0 silk suture. Finally, the intersegmental fissure was separated using three 60-mm (two green and one gold) endostaplers (JJMC) (Fig. 3D).

The specimen was placed in a bag (Cook Medical). Then, a subaortic and paraaortic lymphadenectomy was performed. A wash of the thoracic cavity confirmed the absence of bleeding. The residual lungs were well inflated. Further, a 24-Fr chest drain (Medtronic Company) was placed, and the incisions were closed.

### 2.3 Operative data and hospital course

The operative time was 268 min, and the blood loss was approximately 100 mL. The chest drain was removed 2 days after the surgery, and the postoperative hospital time was 6 days; no complications were noted. The pathological stage was T1aN0M0 stage IA1 as categorized by the UICC TNM Classification (8th edition).

## 3 Discussion

Recently, pulmonary segmentectomy has been frequently performed for early-stage lung cancers or metastatic lung tumors. According to some reports comparing segmentectomy with lobectomy in early-stage lung cancers, no significant difference in the overall and disease-free survival is noted [[2], [3], [4]]. However, in patients with T1 lung adenocarcinoma with spread through air spaces, the risk of any recurrence was significantly higher in the limited resection group than in the lobectomy group [[5], [6], [7]]. Therefore, there is no evidence to strongly recommend performing segmentectomy for early-stage lung cancer. However, segmentectomy can preserve pulmonary function better than lobectomy and can contribute to the maintenance of postoperative quality of life [4]. Therefore, we believe that segmentectomy is a valid alternative to lobectomy, particularly in high-risk operable patients.

VATS is currently a better option than thoracotomy for lung resection; however, VATS segmentectomy is more difficult than thoracotomy [8]. On the left side, upper or lingular segmentectomy is common and easy to perform by VATS. However, depending on the location of the lesion, an additional segment has to be resected using a complicated technique.

VATS segmentectomy is equivalent to VATS lobectomy in terms of the lymphadenectomy result, overall and disease-free survival, 30-day mortality, postoperative complications, and shorter length of hospital stay [9,10]．

Segmentectomy has a risk of locoregional recurrence if a sufficient margin cannot be secured. Furthermore, it is difficult to delineate safe margins for lesions that are not visible and palpable. We usually perform preoperative CT-guided marking in the case of not visible and palpable lesion close to the intersegmental fissure. The preoperative CT-guided marking technique can visualize the location of the lesion. Thus, it is useful for delineating safe margins in VATS segmentectomy for invisible lesions.

## 4 Limitation

The operative time was prolonged due to the complex procedure and confirmation of segmental bronchus by intraoperative bronchoscopy. It is necessary to shorten the operative time to further reduce the invasiveness.

## 5 Conclusion

VATS segmentectomy is safe and reliable and may further broaden the indications for VATS. In this report, we have described specific technical aspects of VATS left anterior and lingular segmentectomy for the impalpable lung nodule using a CT-guided nodule marking prior to the surgery, highlighting the port design and dissection methods for the segmental vessels, bronchus, and intersegmental lung parenchyma. The technique resulted in complete tumor resection with minimal adverse effects.

## Conflict of interest

The author has no conflicts of interest to declare.

## Sources of funding

Authors had no sources of funding.

## Ethical approval

The IRB committee of our hospital decided that approval is not necessary for this case.

## Consent

Written informed consent was obtained from the patient for the publication of this case report and any accompanying images. A copy of the written consent is available for review by the Editor-in-Chief of this journal.

## Author contribution

Kotaro Mizuno: Conceptualization, Methodology, Writing – Original Draft, Writing – Review & Editing, Supervision.

Tadashi Sakane: Writing – Review & Editing.

## Registration of research studies

We haven’t registered our report to the registry system because this report is a case report.

## Guarantor

Kotaro Mizuno.

## Provenance and peer review

Not commissioned, externally peer-reviewed.
